# Acute Encephalopathy and Pandemic (H1N1) 2009

**DOI:** 10.3201/eid1611.100682

**Published:** 2010-11

**Authors:** Song Mi Moon, Sung-Han Kim, Min Hee Jeong, Eun Hye Lee, Tae-Sung Ko

**Affiliations:** Author affiliation: University of Ulsan College of Medicine, Seoul, South Korea

**Keywords:** Encephalopathy, influenza, PCR, pandemic (H1N1) 2009, viruses, letter

**To the Editor:** Since the World Health Organization declared a global pandemic of influenza A pandemic (H1N1) 2009 in June 2009, the number of cases of this strain of influenza has steadily risen. Although most cases have been mild, with complete and uneventful recovery, multiple cases of severe infection with complications, including death, have been reported. Yet the neurologic complications of this virus have been rarely described. We read with interest the article by Kitcharoen et al. (*1*) concerning a patient with encephalopathy associated with pandemic (H1N1) 2009, which progressed to produce quadriplegia with diffuse sensory loss. In that study, however, pandemic (H1N1) 2009 virus was not isolated from the patient’s cerebrospinal fluid (CSF) or brain tissue or detected by reverse transcription–PCR (RT-PCR). We report a case in an adolescent patient with encephalopathy-associated pandemic (H1N1) 2009 that was confirmed by real-time RT-PCR of CSF.

On November 2, 2009, a previously healthy 16-year-old girl was admitted to Asan Medical Center, Seoul, South Korea. Five days earlier, she had sought care for cough, fever (maximum 38.5°C), and mild headache. Enzyme immunoassay (SD Bioline rapid influenza test; Standard Diagnostics Inc., Yongin, South Korea) of a nasopharyngeal swab was positive for influenza virus. Because a large outbreak of pandemic (H1N1) 2009 was concurrent, she was given a presumptive diagnosis and treated with oseltamivir, 75 mg 2×/d, for 5 days. However, her headache worsened, and she was referred to the hospital.

At admission, her temperature was 36.8°C. Examination showed no disturbance of consciousness or focal neurologic deficits except for a severe headache. Results of routine laboratory tests, including serologic tests for HIV, were negative. Real-time RT-PCR of a nasopharyngeal swab at admission was negative for pandemic (H1N1) 2009 virus; a serologic test for this virus was not performed. A magnetic resonance imaging (MRI) scan of the patient’s brain at admission is shown in the Figure, panel A. Examination of CSF showed 0 cells/mm^3^, protein 35.4 mg/dL, glucose 48 mg/dL; blood glucose level was 49%. No bacteria or fungi were isolated from CSF, but pandemic (H1N1) 2009 virus was detected by real-time RT-PCR (Roche Diagnostics, Mannheim, Germany). On the basis of the MRI and RT-PCR results, we diagnosed encephalopathy-associated pandemic (H1N1) 2009 infection. By hospital day 3, her headache and respiratory symptoms had improved, and she was discharged on day 10 without headache or other neurologic signs. A follow-up brain MRI, obtained 1 month later, is shown in the Figure, panel B.

**Figure:**
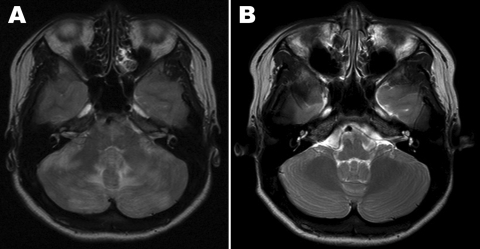
Magnetic resonance imaging (MRI) scans of case-patient’s brain. A) MRI at hospital admission shows ill-defined T2 changes in both cerebellar hemispheres, periventricular white matter, and the pons. B) MRI of the brain 1 month later, showing nearly complete disappearance of the changes observed at admission.

Several hypotheses have been proposed regarding the pathogenesis of influenza-associated acute encephalopathy (IAAE) (*2*): the most straightforward is that it is caused by viral infection of the central nervous system. The isolation of influenza virus from the CSF of living patients (*3*) (or its detection by RT-PCR) and from brain tissue of patients who have died (*4*) supports this hypothesis. More frequently, however, influenza virus has not been detected in the CSF or brains of affected patients despite thorough attempts. Thus, other possible methods for the assessing the pathogenesis of IAAE have been proposed: elevated concentrations of several cytokines such as interleukin (IL)–6, tumor necrosis factor (TNF)–α, and soluble TNF receptor-1; or determination of renal and hepatic dysfunction (*2*). Although IAAE in adults and children was reported during the pandemic (H1N1) 2009 pandemic (*1*,*5*–*8*), this virus was not detectedby virus isolation or RT-PCR in CSF and brain tissue of these patients. The virus was detected in CSF of an infant 3 months of age with IAAE (*8*); however, the virus may have been found in CSF because of the presence of blood from a traumatic lumbar puncture.

The absence of pleocytosis and the normal protein and glucose levels in CSF from the patient described here were noteworthy. Previous reports showed that leukocyte counts within normal limits (70%–90%) were found in CSF of patients with IAAE and seasonal influenza infection (*9**,**10*). Recent publications on IAAE and concurrent pandemic (H1N1) 2009 virus infection also reported no increase in CSF leukocyte count and protein level (*1*,*5*–*7*). Therefore, absence of CSF pleocytosis and protein levels within normal limits are common with IAAE.

The diagnosis of IAAE in the patient reported here is probable, based on positive real-time RT-PCR results from CSF examination and brain MRI findings. However, some limitations should be mentioned. First, a positive RT-PCR result could have resulted from contamination associated with clinical procedures and laboratory assays. Nonetheless, we believe that the lumbar puncture was done aseptically and that the real-time RT-PCR performed in the hospital’s clinical microbiologic laboratory was reliable. Second, the brain MRI findings were also nonspecific and could be associated with hypoxia, edema, or other unknown processes. However, the patient had no history of hypotensive episodes, hypoxemia, abnormal metabolic and toxic processes, and other infectious disease. In conclusion, IAAE with pandemic (H1N1) 2009 may be caused by direct viral infection of the CNS and, although its pathogenesis is not clear, physicians should remain alert to this possibility.
